# Collagenolytic Activity Is Associated with Scar Resolution in Zebrafish Hearts after Cryoinjury

**DOI:** 10.3390/jcdd4010002

**Published:** 2017-02-24

**Authors:** Laurent Gamba, Armaan Amin-Javaheri, Jieun Kim, David Warburton, Ching-Ling Lien

**Affiliations:** 1 Heart Institute of Children’s Hospital Los Angeles, 4661 Sunset Blvd, Los Angeles, CA 90027, USA; aminjava@usc.edu (A.A.-J.); jieunbucks@gmail.com (J.K.); dwarburton@chla.usc.edu (D.W.); 2 Saban Research Institute of Children’s Hospital Los Angeles, Program of Developmental Biology and Regenerative Medicine, 4661 Sunset Blvd, Los Angeles, CA 90027, USA; 3 Department of Surgery, Keck School of Medicine, University of Southern California, 4661 Sunset Blvd, Los Angeles, CA 90027, USA; 4 Department of Biochemistry & Molecular Medicine, Keck School of Medicine, University of Southern California, 4661 Sunset Blvd, Los Angeles, CA 90027, USA

**Keywords:** heart regeneration, in situ zymography

## Abstract

Myocardial infarction is the major cause of cardiac injury in western countries and can result in a massive loss of heart cells, leading eventually to heart failure. A fibrotic collagen-rich scar may prevent ventricular wall rupture, but also may result in heart failure because of its stiffness. In zebrafish, cardiac cryoinjury triggers a fibrotic response and scarring. Unlike with mammals, zebrafish heart has the striking ability to regenerate and to resolve the scar. Thus, understanding the mechanisms of scar resolution in zebrafish heart might facilitate the design of new therapeutic approaches to improve the recovery of patients. To visualize the collagenolytic activity within the zebrafish heart following cryoinjury, we used an in situ collagen zymography assay. We detected expression of *mmp2* and *mmp14a* and these matrix metalloproteinases might contribute to the collagenase activity. Collagenolytic activity was present in the wound area, but decreased as the myocardium regenerated. Comparison with neonatal mouse hearts that failed to regenerate after transmural cryoinjury revealed a similar collagenolytic activity in the scar. These findings suggest that collagenolytic activity may be key to how the zebrafish heart resolves its scar; however, it is not sufficient in mouse hearts that lack efficient myocardial regeneration.

## 1. Introduction

Myocardial infarction is the most common cause of heart injury in humans and results in acute loss of a large number of cardiac cells. In mammals, the heart is unable to regenerate the lost cardiomyocytes after myocardial infarction. Instead, the injury triggers the activation of fibroblasts that secrete collagen that patches the heart and prevents the wall from rupturing. However, this collagen-based scar persists in the heart and contributes to abnormal systolic function due to its stiffness and non-contractibility and eventually heart failure can occur [1]. Unlike mammals, the zebrafish has a remarkable ability to regenerate its heart after ventricular amputation, mainly by the ability of the remaining cardiomyocytes to de-differentiate and proliferate to replace the lost cardiac tissue [2,3]. Recently, a cryoinjury model that more closely mimics the pathophysiological process undergone by the human heart after myocardial infarction was established in zebrafish [4,5]. Following cryoinjury, a collagen scar forms at 14–21 days post-cryoinjury (dpc), and the zebrafish heart is able to resolve the scar concomitantly to myocardial regeneration, which mammals cannot do.

Smad3-dependent TGFβ signaling plays an important role in myocardial infarction-induced fibrosis and cardiac remodeling in mammals [6]. Chablais and colleagues showed that the balance between the reparative and regenerative processes is achieved through Smad3-dependent TGFβ signaling, and that this signaling is important for the formation of the transient scar. This demonstrates that the zebrafish and mammals share similar mechanisms of scar formation [7]. Interestingly, the hearts that fail to form this transient scar also fail to undergo cardiomyocyte proliferation and myocardial regeneration. As a result, these hearts form a bulging ventricle, suggesting that scar formation is a necessary step required for heart regeneration following cryoinjury in fish [7]. However, the mechanisms underlying collagen scar resolution remain unsolved. Understanding the mechanisms of scar resolution in zebrafish would allow us to design new therapeutic approaches to reduce collagen scars in patients suffering from myocardial infarction-induced heart failure.

In this study, we set forth to determine the mechanisms involved in collagen degradation and scar resolution by characterizing the collagenolytic activity in the zebrafish heart during regeneration following cryoinjury. To do so, we used, for the first time in adult zebrafish hearts, an in situ collagen zymography assay that allows the localization of collagenolytic activity on tissue sections. Following cryoinjury, strong collagenolytic activity was detected in the wound/regenerating area, and this activity decreases as the scar is being resolved. We observed a similar pattern of collagenolytic activity and collagen expression, suggesting that a balance of collagen synthesis and degradation takes place in the wound area. Transcripts of matrix metalloproteinase (*mmp*) genes, *mmp2* and *mmp14*, and MMP2 enzymatic activity are increased following cryoinjury, suggesting that they are the potential proteases involved in collagen degradation. Lastly, we observed collagenolytic activity in the wound area of neonatal mouse hearts, similar to that of fish hearts. These results suggest that the scar resolution in regenerating zebrafish hearts is at least partially achieved by collagenase activities. However, collagenase activities are not sufficient to resolve scars in mouse hearts after severe cryoinjury, most likely due to insufficient myocardial regeneration.

## 2. Materials and Methods

### 2.1. Animal Procedure

Zebrafish used in this study were maintained using standard procedure [8]. Commercial farm wild-type zebrafish (Aquatica Biotech, Sun City Center, FL, USA) between 4 and 18 months of age were used in this study. One-day-old neonatal mice (ICR/CD-1 strain, Charles River Laboratories, Wilmingon, MA, USA) were used and animals were housed at the Children’s Hospital Los Angeles animal facility. The cryoinjury was performed as described in [5] in zebrafish and [9] in neonatal mice. All experimental procedures on animals have been approved by Children’s Hospital Los Angeles IACUC.

### 2.2. In Situ Collagen Zymography and Immunostaining

Unfixed hearts were incubated overnight at 4 °C in sucrose 30%/PBS, and embedded in Tissue Freezing Medium (Triangle Biomedical Sciences, Inc., Durham, NC, USA). 7-μm thick sections were made with a cryostat (Leica Biosystems, Wetzlar, Germany) and stored at −80 °C until use. For the zymography assay, embedding medium was washed twice in PBST, the sections were pre-incubated for 5 min in the reaction buffer and then incubated 1 h at room temperature in a closed box with DQ collagen type I-fluorescein (D-12060, Molecular Probes, Eugene, OR, USA) diluted 10 times in the reaction buffer using the EnzChek^®^ Gelatinase/Collagenase Assay Kit (E-12055, Molecular Probes). The degradation of the DQ collagen is visualized in situ by the green fluorescence caused by the release of fluorescein conjugates. For immunostaining, the sections were washed in PBST twice and incubated with cold acetone (−20 °C) for 7 min, and washed again with PBST. Sections were then blocked for 1 h at room temperature and incubated overnight at 4 °C with primary antibody. The second day, the sections were washed three times with PBST, incubated with the secondary antibody for 1 h at room temperature and washed three times in PBST and mounted in Vectashield medium with Dapi (H-1500, Vector Laboratories, Inc., Burlingame, CA, USA). Mouse anti-tropomyosin primary antibody (CH1, Developmental Studies Hybridoma Bank, Iowa City, IA, USA) was used at a 1:200 dilution.

### 2.3. Acid Fuschin Orange G (AFOG) Staining

The wound area was visualized by AFOG staining as described in [2]. The cryosections were fixed in PFA 4%/PBS for 30 min prior to the procedure.

### 2.4. In Situ Hybridization

Digoxigenin labeled RNA probes for *col1a1a* (cb21 clone, ZIRC), *mmp2* (forward primer TGGATAACCGTATTACCGCC; reverse primer CGCGCAATTAACCCTCACTAAAGCACTAGTCATACCAGGATC) and *mmp14a* [10] were generated using T7 and T3 RNA polymerases (Promega). Color in situ hybridization was performed as described in [11]. Fluorescent in situ hybridization was performed using the TSA Plus Cyanine 3 System kit (PerkinElmer, Waltham, MA, USA).

### 2.5. RT-Quantitative PCR

Total RNA from six to eight ventricles collected at different time points after injury was extracted using TRIzol^®^ reagent (Thermo Fisher Scientific, Waltham, MA, USA). cDNA was synthesized using Super-Script^®^ III First-Strand synthesis kits (Invitrogen, Carlsbad, CA, USA). Real-Time quantitative PCR was carried out using the Roche LightCycler^®^ 480 Real-Time PCR System, in a 10 μL volume with 1 μM gene specific primers using LightCycler^®^ 480 SYBR Green I Master (Roche, Basel, Switzerland). *eef1a1a* gene was used as a reference gene for normalization and to ensure cDNA integrity. Every PCR reaction was done in triplicate and analyzed with LightCycler^®^ 480 software (Roche). One-way ANOVA followed by a Dunnett’s multiple comparisons test was used to determine statistical significance of the differences between uninjured hearts and hearts at each time point using GraphPad Prism 7 Software (* *p* < 0.05, ** *p* < 0.01,**** *p* < 0.0001, GraphPad Software, Inc., La Jolla, CA, USA). The primer sequences used in this study are the following:

*mmp2* (forward CCTCACCCATCATAAAGTTCC, reverse TTTCTTCAGCGTGTCCTTCAA)
*mmp9* (forward CACCGTTGATGCCATGA, reverse TCCATGTCTGGCGAATAG)
*mmp13a* (forward GAGGCTCAAGGAGATGC and reverse GTTGAGTAGGCCTTGATGT)
*mmp13b* (forward GCAGAAGATCAGAGAGATGC and reverse AGAATCCTGAAGGTCACG)
*mmp14a* (forward CGGGACCAGTGACAAAG, reverse CGAGATAGCGGAGTTGATAGA)
*mmp14b* (forward ACAGTAACAAAGTGGTGTCA, reverse ATACTGCTGTAGCCATGC)
*eef1a1a* (forward CTACAAATGCGGTGGAATC, reverse AATTTCCAGAGAGCAATGTCA)

### 2.6. SDS-PAGE Gelatin Zymography

Aliquots of ventricle lysates (eight ventricles per group) containing 40 μg of proteins were loaded on 0.1% SDS, 8% polyacrylamide gels containing 1% gelatin. After electrophoresis, gels were incubated overnight at 37 °C in substrate buffer (50 mM Tris-HCl pH 7.4 and 10 mM CaCl_2_), stained with 0.25% Coomassie brilliant blue, and destained in methanol/acetic acid/water (50:10:40). Gelatinolytic activity was evident as cleared regions on a dark background. The gel was scanned and the intensity of active MMP2 and pro-MMP2 gelatinolytic bands was measured with ImageJ software.

### 2.7. Confocal Imaging of Heart Sections

Imaging of sections was performed using confocal microscopy (Zeiss LSM710, Carl Zeiss, Oberkochen, Germany). Fluorescence background was removed using the lambda mode of the ZEN software (Zeiss).

## 3. Results

### 3.1. Cryoinjury Induces Extensive Collagenolytic Activity and col1a1a Expression in the Wound Area in Zebrafish Hearts

**Figure 1:**
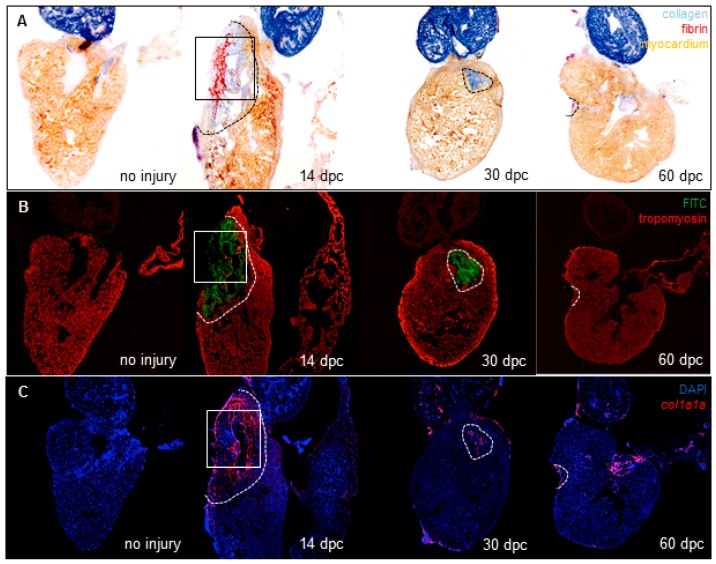

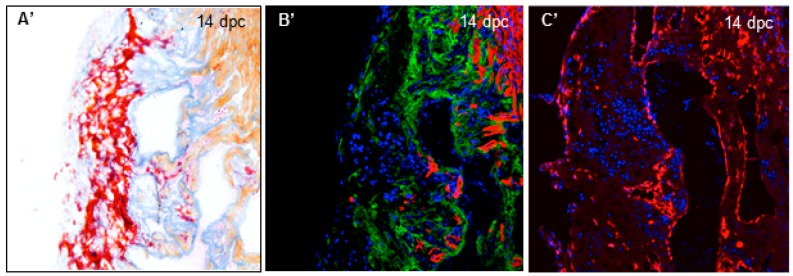
Collagenolytic activity and fibroblast activation during heart regeneration. (**A**) Wound area visualized with AFOG staining of frozen sections from uninjured hearts (*n* = 3) or hearts at 14 dpc (*n* = 7), 30 dpc (*n* = 8) and 60 dpc (*n* = 6) (fibrin in red, collagen in blue, and counterstaining in orange); (**B**) Confocal imaging of consecutive sections from hearts stained in (**A**). Green fluorescence represents the release of fluorescein activity due to the degradation of DQ-collagen substrate. Tropomyosin immunostaining is used as counterstaining (red fluorescence); (**C**) *col1a1a* expression by fluorescent in situ hybridization on consecutive sections from the same hearts shown in (**A**,**B**). *col1a1a* mRNA in red, nuclei in blue (DAPI). Comparison of collagenolytic activity (**B’**) and fibroblasts (**C’**) with collagen (**A’**) on consecutive sections of the 14 dpc heart at a 20× magnification (magnification of the boxes in (**A**–**C**) respectively). Dashed lines represent the wound area.

We hypothesized that collagen scars are degraded by collagenases during zebrafish heart regeneration after cryoinjury. To characterize this process, a cryoinjury model was created by placing a probe pre-cooled in liquid nitrogen on the ventricle as described [5]. In order to visualize the collagenase activity spatially, we performed in situ collagen zymography assays on zebrafish hearts. We used unfixed frozen hearts in order to maintain the functionality of the enzymes. Heart sections were incubated with a type I-collagen conjugated to fluorescein. The collagenolytic activity releases fluorescein conjugates that appear in the extracellular space surrounding the cells that degrade the collagen. We chose to examine hearts at 14 days post-cryoinjury (dpc) because in our hands, cryoinjury-induced collagen scars form between 7 and 14 dpc and begin to decrease from 14 dpc and no or very little collagen is observed at 60 dpc (Figure 1A). While no detectable fluorescence is observed in uninjured hearts (*n* = 3), extensive fluorescence is observed in the wound area of injured hearts (*n* = 7) at 14 dpc, indicating that collagenolytic activity is induced in response to the injury (Figure 1B). The area with fluorescence decreases at 30 dpc (*n* = 8) and 60 dpc (*n* = 6) as the myocardium regenerates (Figure 1B). Collagen localization was further analyzed with a higher magnification at 14 dpc, revealing that collagen is mostly within and surrounding the wound area (Figure 1A’). This result suggests that these cell types might be one of the potential sources from which collagen is secreted. Interestingly, in situ zymography performed on a consecutive section of the same heart shows that the collagenolytic activity is present in the same areas as the collagen (Figure 1B’), suggesting that the cells involved in the degradation of the collagen are either the ones that secrete the collagen or are closely associated with them.

Following myocardial infarction, the fibroblasts are activated and secrete large amounts of type I and III collagens in order to replace the lost myocardium and provide a mechanical support to prevent the wall from rupturing [12]. In order to visualize the fibroblasts in the zebrafish heart, we used the *col1a1a* gene encoding the collagen type I alpha 1 as a cardiac fibroblast marker [13] and we performed fluorescent in situ hybridization on consecutive sections of hearts used in Figure 1A. In uninjured hearts, there is no detectable expression of *col1a1a*. *col1a1a* expression is induced after injury in the wound area and the number of *col1a1a+* cells in the wound decreases as the scar is being resolved (Figure 1C). Interestingly, *col1a1a* is expressed in the wound, likely in the infiltrated cells and cell populations surrounding the wound at 14 dpc (Figure 1C’) and matches the collagen pattern in AFOG staining in Figure 1A’, as expected.

### 3.2. Matrix Metalloproteinases Transcripts Increase as well as MMP2 Activity Following Cryoinjury in Zebrafish Hearts

**Figure 2:**
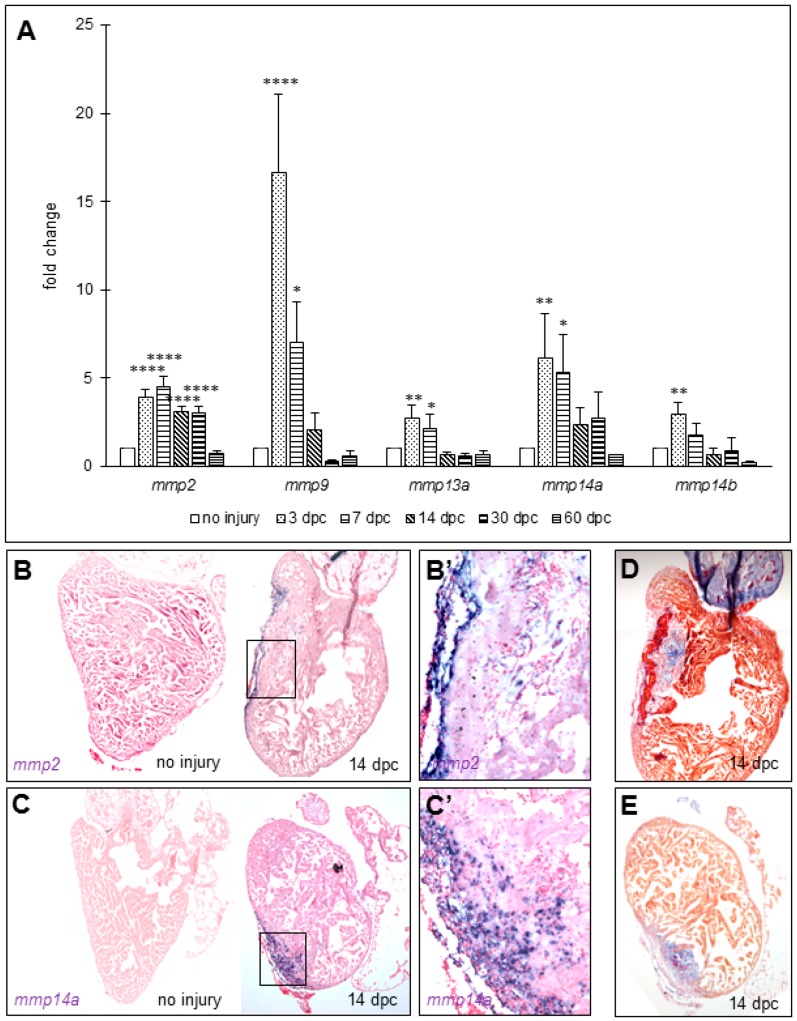
MMPs transcripts increase in zebrafish ventricles after cryoinjury. (**A**) RT-quantitative PCR for *mmp2*, *mmp9*, *mmp13a*, *mmp14a*, and *mmp14b* in ventricles at several time points after cryoinjury (six to eight ventricles per time point). Significant difference with uninjured hearts: **** *p* < 0.0001, ** *p* < 0.01, * *p* < 0.05; (**B**,**C**) in situ hybridization in uninjured and 14 dpc hearts of *mmp2* (*n* = 1 and 2, respectively) and *mmp14a* (*n* = 2 and 2, respectively) transcripts respectively; (**B’**,**C’**) Magnification of the boxes in (**B**,**C**), respectively; (**D**,**E**) AFOG staining on consecutive sections of 14 dpc hearts shown in (**B**,**C**) respectively for comparison with the wound area (fibrin in red, collagen in blue, and counterstaining in orange).

Since there are no genes encoding type-III collagens in zebrafish [14], we focused on the enzymes that can lyse type-I collagens. Such enzymes are named type-I collagenases and belong to the matrix metalloproteinase (MMP) family. In order to identify the potential MMPs involved in the degradation of the collagen during scar resolution, we screened genes encoding type I collagenases (*mmp13a/b* and *mmp14a/b*) and gelatinases (*mmp2* and *mmp9*) that are expressed in the zebrafish heart following cryoinjury by RT-quantitative PCR. It is noteworthy to mention that there are no zebrafish homologs for the type I collagenases MMP1 and 8 [15]. Our data show an increase of the transcripts for all the MMPs at 3 dpc compared to uninjured hearts, except for *mmp13b* of which transcripts are not detected (Figure 2A). Transcript levels of *mmp9*, *13a*, and *14b* decrease at 7 dpc and go back to the basal levels at 14 dpc, suggesting a role in the early phase of the response to the injury, such as inflammatory response or scarring. However, *mmp2* transcripts decrease between 7 and 14 dpc but remain more elevated than basal levels at 14 and 30 dpc (fold increase 3.11 ± 0.31 and 3.05 ± 0.31, *p* < 0.0001 at 14 and 30 dpc respectively). Although *mmp14a* transcripts are not significantly higher at 14 and 30 dpc in our RT-quantitative PCR assay (fold increase 2.36 ± 0.96 and 2.71 ± 1.54 at 14 and 30 dpc respectively), there is a trend that its expression is persistent at these time points compared to *mmp9, mmp13a*, and *mmp14b*. In order to confirm the persisting expression of *mmp2* and *mmp14a*, we performed an in situ hybridization to visualize the expressions of *mmp2* and *mmp14a* in the heart at 14 dpc. Our data reveal that *mmp2* and *mmp14a* transcripts are both present specifically in the wound area, visualized with AFOG staining, while there is no detectable staining in uninjured hearts (Figure 2B–E). Magnification of the wound area at 14 dpc shows that *mmp2* and *mmp14a* are strongly expressed in the wound and surrounding cells (Figure 2B’,C’).

**Figure 3:**
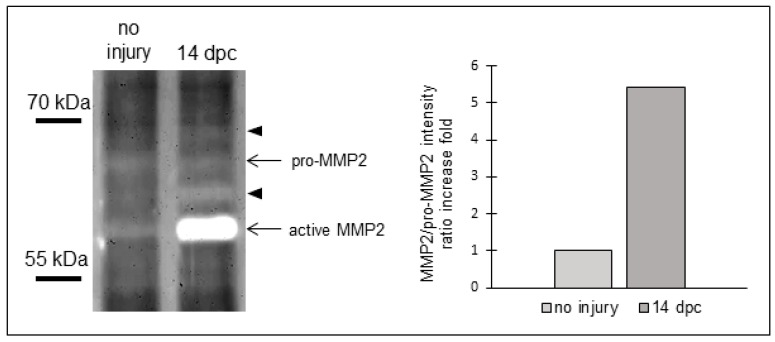
MMP2 activity increases in zebrafish ventricles after cryoinjury. Gelatin zymography in uninjured and 14 dpc hearts (eight ventricles per condition). Left panel: Zymogram showing gelatinolytic bands for pro and active MMP2 and additional gelatinolytic bands for undetermined MMPs (arrowheads); Right panel: MMP2/pro-MMP2 intensity ratio.

However, an increase of transcripts does not necessarily correlate with the enzymatic activity of these MMPs. MMP2 is synthesized as an inactive pro-MMP2 and its cleavage by proteases leads to a shorter, enzymatically active form [16]. To assess MMP2 activity, we performed a gelatin zymography that allows us to detect the two forms of MMP2 (pro-MMP2 and active MMP2). MMP2 zymogen bands revealed a pronounced increase in the intensity of the active form of MMP2 (lower band) at 14 dpc compared to uninjured hearts (Figure 3), showing that MMP2 activity increases as well. In addition, the band size of pro-MMP2 is lower than its predicted size (approximatively 67 kDa and approximatively 72 kDa, respectively), similarly to what was described in osteoclasts during regeneration of zebrafish scales [17]. We also observed additional gelatinolytic bands with stronger intensities at 14 dpc compared to uninjured hearts, suggesting that other MMPs are active at 14 dpc and may be involved in the scar regression as well. Taken together, these data suggest that MMP2 and MMP14 are likely involved in the degradation of the extracellular matrix (ECM) components during the scar resolution process, and in the collagenolytic activity observed in Figure 1.

### 3.3. Collagenolytic Activity Is Present in the Scar Area of Neonatal Mouse Hearts Following Cryoinjury

**Figure 4:**
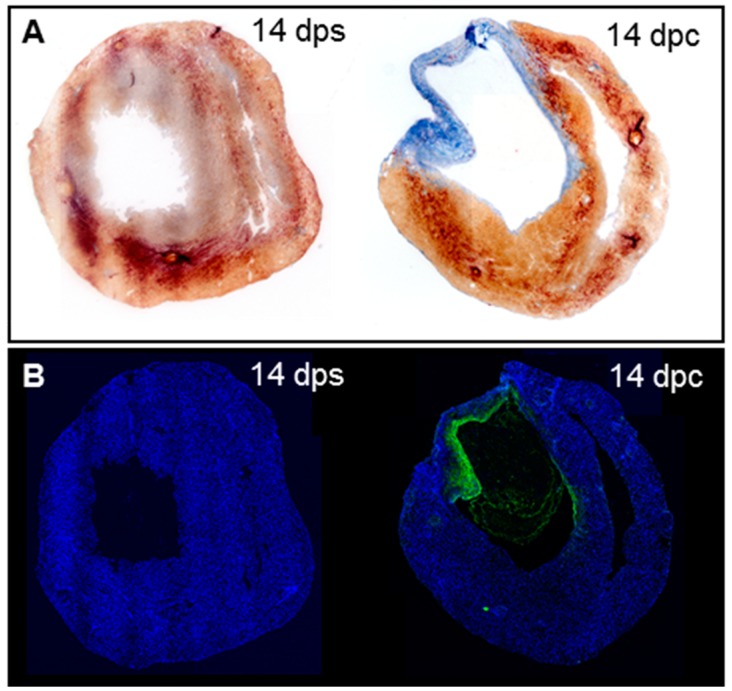
Collagenolytic activity in the scar of neonatal mouse hearts after cryoinjury. (**A**) AFOG staining on neonatal mouse hearts at 14 dps (*n* = 3) and 14 dpc (*n* = 4); (**B**) In situ zymography on consecutive sections from hearts in (**A**).

We found that strong collagenolytic activity is present in the wound area after cryoinjury, consistent with previous studies reporting that zebrafish hearts are able to resolve the collagen scar during regeneration. While the neonatal mouse heart regenerates its myocardium after ventricle amputation [18], it does not fully regenerate after transmural cryoinjury and the collagen scar is not resolved [9]. Thus, we set out to determine whether the collagen degradation defect in the neonatal mouse heart can be explained by an absence of collagenolytic activity. To assess this question, we performed in situ collagen zymography in neonatal mouse hearts that have been severely cryoinjured. Massive collagen deposition occurred in the wound area of neonatal mouse hearts at 14 days post-transmural cryoinjury while no collagen is deposited in the ventricular wall of sham-operated hearts as visualized by the absence of blue staining after AFOG staining (Figure 4A), as previously reported [9]. In situ collagen zymography on consecutive cryosections reveals that no fluorescence is observed in the sham-operated hearts (*n* = 3), however, collagenolytic activity is present on the internal side of the injured cardiac wall and in the endocardium of the ventricular chamber (*n* = 4, Figure 4B). This result shows that the injury induces collagenolytic activity that can degrade collagen inside the wound, similarly to the zebrafish heart.

## 4. Discussion

Following an injury, cell death, inflammatory infiltration, and increased mechanical loads lead to the activation of fibroblasts, which proliferate, transdifferentiate into myofibroblasts and increase secretion of ECM components, mostly collagen, in the wound area [1,19,20]. This excess of ECM components deposition, called fibrosis, is necessary to maintain the integrity of the cardiac wall following the death of muscle cells. In the meantime, cardiac cells and neutrophils secrete MMPs, leading to the degradation of the ECM, cardiac remodeling and ventricular dilatation and eventually to heart failure [12,21]. Particularly, MMP14 is secreted by cardiomyocytes and fibroblasts after myocardial infarction and participates to myocardial remodeling [22,23]. Consistently, our data show that a strong collagenolytic activity is present in the injured cardiac wall of the neonatal mouse heart, likely involved in the cardiac remodeling. Collagenolytic activity is present in areas where collagen is deposited, suggesting that a balance between collagen synthesis and degradation occurs at the site of injury in both fish and neonatal mouse hearts. Since we observe a similar pattern of collagen expression and collagenolytic activity in the wound of both zebrafish and neonatal hearts, the collagenolytic activity alone is not sufficient to explain how the scar is eventually resolved in fish hearts.

**Figure 5:**
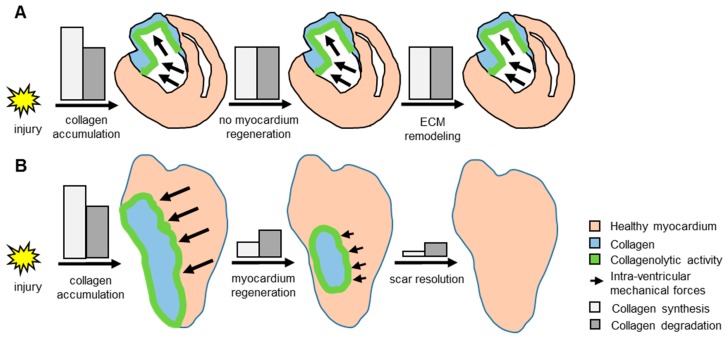
Model of potential scar resolution mechanism in zebrafish heart following injury. (**A**) In the neonatal mouse heart after transmural cryoinjury, damage to the hearts triggers both collagen synthesis and collagenolytic activity in the wound. The loss of cardiac muscle increases the intra-ventricular mechanical forces (represented by black arrows) which stimulates the synthesis of collagen. The absence of myocardium regeneration leads to a balance of intra-ventricular mechanical forces and of collagen synthesis/degradation, resulting in persisting collagen scar, and ECM remodeling; (**B**) In zebrafish, the injury induces a similar fibrotic response. However, when the myocardium is regenerating, the intra-ventricular mechanical forces decrease, leading to less collagen synthesis than collagen degradation, and eventually to the scar resolution.

To explain how the scar is resolved in fish hearts and not in neonatal mouse hearts, we propose the following model (Figure 5). The mechanical loads in fish hearts decrease as the myocardium regenerates, leading to less collagen deposition. The balance between collagen synthesis and degradation shifts towards more degradation than synthesis, leading eventually to complete collagen disappearance. In the neonatal mouse heart after transmural cryoinjury, no myocardium regeneration occurs. Therefore, the mechanical loads are maintained, and the fibroblasts keep producing collagen. Thus, the scar resolution would be a process dependent on the regeneration of the myocardium. This hypothesis is supported by a recent study reporting that hearts from *tert* mutant zebrafish failed to regenerate and to resolve their scar following cryoinjury [24]. If the collagen degradation is a process independent of the renewal of the myocardium, we would expect to observe a bulging ventricle because of the lack of both scar and myocardium, similarly to what was observed in fish that fail to form a scar following cryoinjury [7].

Our data suggest that MMP2 and MMP14 are likely involved in the degradation of the collagen during scar resolution because they are the two collagenases which elevated expression is maintained throughout the scar resolution process. Following cryoinjury, type-I collagen is secreted in the wound area in zebrafish hearts as indicated by the increase of *col1a1a* transcripts visualized by in situ hybridization. Out of the four known mammalian type-I collagenases—including MMP1, 8, 13, and 14—only MMP13 and MMP14 have a zebrafish counterpart [15]. Our RT-quantitative PCR screening shows that only MMP14a has increased level of transcription throughout the whole scar resolution process, indicating that this MMP is very likely the one involved in the degradation of the type-I collagen. However, only loss of function experiments using inducible tissue-specific zebrafish transgenic line carrying a dominant negative form of MMP14 will allow definitive identification of the specific role of MMP14 during the scar resolution. We showed that the active form of MMP2 is increased following cryoinjury, supporting a role of MMP2 in the scar resolution process. MMP2 is an enzyme known to degrade the gelatin, an intermediate product of type-I collagen degradation. However, several reports showed evidence of MMP2 as a type-I collagenase, similarly to interstitial collagenases [25,26]. For example, MMP2 is important for the migration of lymphatic endothelial cells through a fibrillar type-I collagen matrix in both mice and zebrafish [27]. Thus, MMP2 may be involved in the degradation of mature type-I collagen fibers and/or the gelatin. Interestingly, MMP14 was reported as one of the proteases capable to cleave MMP2 into its active form [28] and may be one of the MMP2 activators in the wound. Additionally, in our gelatin zymography assay we observed undetermined zymogenic bands, suggesting that other MMPs are active at 14 dpc and may be involved in scar resolution as well.

Our data indicate that following injury, cells surrounding the wound can express *col1a1a* transcripts and these cell populations might adopt a fibroblast-like behavior in zebrafish hearts. These cells are likely to be endocardial and epicardial cells. This is consistent with a previous report that epicardium expresses fibroblast marker genes following cryoinjury in fish hearts, such as vimentin and tenascin-C [5]. Particularly, Gonzalez-Rosa and colleagues suggested that epicardium-derived cells (EPDCs) transdifferentiate into myofibroblasts following cryoinjury and express the fibroblast markers *col1a2* and *periostin*, undergoing epithelial-mesenchymal transition and migrating towards the wound area [29].

In mice, using cre/lox lineage tracing experiments, two recent studies reported that resident fibroblasts were the main cell population to transdifferentiate into myofibroblasts and both papers excluded the epicardium as a significant provider of myofibroblasts upon heart injury [13,30]. In fish, it therefore appears that the mechanism of fibrosis might be different from mice. In mice, resident fibroblasts are activated upon injury, migrate towards the injury site and transdifferentiate into myofibroblasts to synthesize the collagen to protect the heart, while in fish epicardium/endocardium can also fulfill this function although we cannot rule out that resident fibroblasts also contribute to fibrosis. However, whether endocardium and endocardium derived cells transdifferentiate into myofibroblasts or adopt a fibroblast-like behavior remains to be further determined. In fish, following cryoinjury, EPDCs express fibroblast markers and migrate towards the injury site [29], and to this regard, fish epicardium and murine resident fibroblasts share a similar function in response to the injury. Interestingly, the murine resident fibroblasts participating to the injury-induced fibrosis are mostly from embryonic epicardium lineage [13,30]. However, no studies reported that endocardium contributes to the fibrosis in zebrafish hearts so far. Our data show that endocardium might also express *col1a1a* in response to the injury and then participate in the fibrotic response in fish hearts. This is another difference from the mouse heart since endothelial cells do not transdifferentiate into myofibroblasts following injury [13,30].

## 5. Conclusions

Our results show that cryoinjury induces collagenolytic activity and collagen synthesis at the same location in zebrafish hearts, indicating that a collagen turn-over takes place in the wound area, similarly to what occurs in murine hearts. We observed that this collagenolytic activity decreases as the myocardium is regenerating. These observations, combined with what we already know of the post-myocardial infarcted mammalian hearts from the literature, led us to propose a model to explain how the scar is resolved in fish hearts and not in mammalian hearts following injury. We propose that the scar is resolved, at least for a part, as a consequence of the regeneration of the myocardium, occurring only in fish.
